# Borgs are giant genetic elements with potential to expand metabolic capacity

**DOI:** 10.1038/s41586-022-05256-1

**Published:** 2022-10-19

**Authors:** Basem Al-Shayeb, Marie C. Schoelmerich, Jacob West-Roberts, Luis E. Valentin-Alvarado, Rohan Sachdeva, Susan Mullen, Alexander Crits-Christoph, Michael J. Wilkins, Kenneth H. Williams, Jennifer A. Doudna, Jillian F. Banfield

**Affiliations:** 1 Innovative Genomics Institute, University of California, Berkeley, CA USA; 2 Department of Plant and Microbial Biology, University of California, Berkeley, CA USA; 3 Environmental Science, Policy and Management, University of California, Berkeley, CA USA; 4 Earth and Planetary Science, University of California, Berkeley, CA USA; 5 Department of Soil and Crop Sciences, Colorado State University, Fort Collins, CO USA; 6 Lawrence Berkeley National Laboratory, Berkeley, CA USA; 7 Rocky Mountain Biological Lab, Gothic, CO USA; 8 Department of Chemistry, University of California, Berkeley, CA USA; 9 The University of Melbourne, Melbourne, Victoria Australia

**Keywords:** Metagenomics, Climate-change mitigation

## Abstract

Anaerobic methane oxidation exerts a key control on greenhouse gas emissions^1^, yet factors that modulate the activity of microorganisms performing this function remain poorly understood. Here we discovered extraordinarily large, diverse DNA sequences that primarily encode hypothetical proteins through studying groundwater, sediments and wetland soil where methane production and oxidation occur. Four curated, complete genomes are linear, up to approximately 1 Mb in length and share genome organization, including replichore structure, long inverted terminal repeats and genome-wide unique perfect tandem direct repeats that are intergenic or generate amino acid repeats. We infer that these are highly divergent archaeal extrachromosomal elements with a distinct evolutionary origin. Gene sequence similarity, phylogeny and local divergence of sequence composition indicate that many of their genes were assimilated from methane-oxidizing *Methanoperedens* archaea. We refer to these elements as ‘Borgs’. We identified at least 19 different Borg types coexisting with *Methanoperedens* spp. in four distinct ecosystems. Borgs provide methane-oxidizing *Methanoperedens* archaea access to genes encoding proteins involved in redox reactions and energy conservation (for example, clusters of multihaem cytochromes and methyl coenzyme M reductase). These data suggest that Borgs might have previously unrecognized roles in the metabolism of this group of archaea, which are known to modulate greenhouse gas emissions, but further studies are now needed to establish their functional relevance.

## Abstract (web summary)

Borgs are remarkably large, divergent archaeal extrachromosomal elements with metabolic genes linked to the methane cycle.

## Main

Of all of the biogeochemical cycles on Earth, the methane cycle may be most tightly linked to climate. Methane (CH_4_) is a greenhouse gas roughly 30 times more potent than carbon dioxide (CO_2_), and approximately 1 gigatonne is produced annually by methanogenic (methane-producing) archaea that inhabit anoxic environments^2^. The efflux of methane into the atmosphere is mitigated by methane-oxidizing microorganisms (methanotrophs). In oxic environments, CH_4_ is consumed by aerobic bacteria that use methane monooxygenase (MMO) and O_2_ as a terminal electron acceptor^3^, whereas in anoxic environments, anaerobic methanotrophic archaea (ANME) use a reverse methanogenesis pathway to oxidize CH_4_, the key enzyme of which is methyl-CoM reductase (MCR)^4,5^. Some ANMEs rely on a syntrophic partner to couple CH_4_ oxidation to the reduction of terminal electron acceptors, yet *Methanoperedens* (ANME-2d, phylum Euryarchaeota) can directly couple CH_4_ oxidation to the reduction of iron, nitrate or manganese^6,7^. Some phenomena have been suggested to modulate rates of methane oxidation. For example, some phages can decrease rates of methane oxidation by infection and lysis of methane-oxidizing bacteria^8^, and others with the critical subunit of MMO^9^ probably increase the ability of their host bacteria to conserve energy during phage replication. Here we report the discovery of novel extrachromosomal elements (ECEs) that are inferred to replicate within *Methanoperedens* spp. Their numerous and diverse metabolism-relevant genes, huge size and distinctive genomic architecture distinguish these archaeal ECEs from all previously reported elements associated with archaea^10–12^ and from bacteriophages, which typically have one or a few biogeochemically relevant genes^13,14^. We hypothesize that these novel ECEs may substantially impact the capacity of *Methanoperedens* spp. to oxidize methane.

## Genome structure and features

**Fig. 1:**
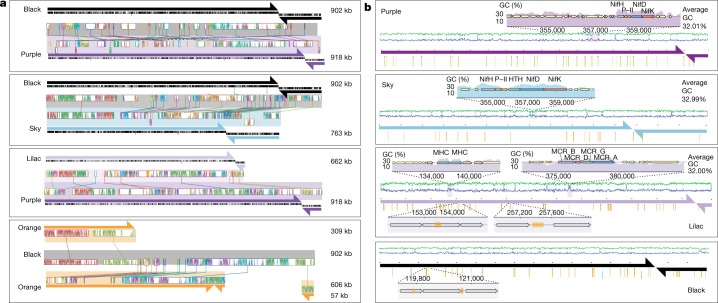
Borgs share overall genomic features. **a**, Genome replichores (arrows) and coding strands (black bars) for aligned pairs of the four complete (Black, Purple, Sky and Lilac) and one near-complete (Orange) Borg. Blocks of sequence with identifiable nucleotide similarity are shown in between each pair (coloured graphs linked by lines; *y* axes show similarity). **b**, Genome overviews showing the distribution of three or more perfect tandem direct repeats (gold rods) along the complete genomes. Insets provide examples of local elevated GC content associated with certain gene clusters and within gene and intergenic tandem direct repeats (gold arrows).

**Fig. 2:**
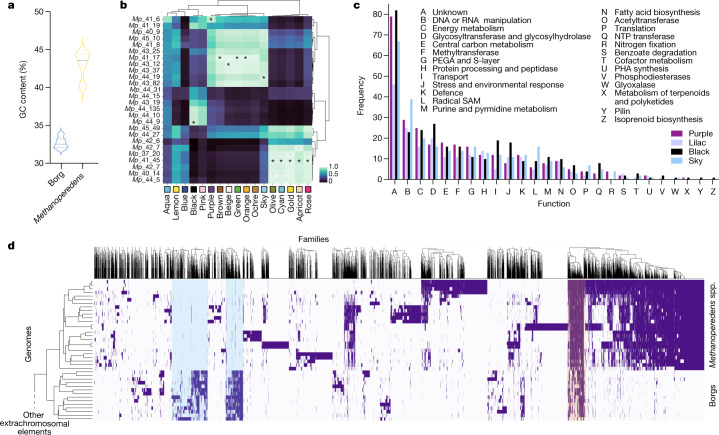
Borg and *Methanoperedens* spp. genomic features and abundance patterns. **a**, The average genome GC contents of Borgs and *Methanoperedens* spp. are distinct. The black line denotes the median, and the dashed lines show the interquartile range. **b**, Groups of related *Methanoperedens* spp. (rows) correlate with groups of Borgs (columns) across a set of 50 samples. The asterisks indicate two-sided Pearson correlations above 0.92 with FDR-corrected *P* values below 2.0 × 10^–20^ that suggest that Brown, Green, Orange, Beige and Ochre Borgs associate with one group of *Methanoperedens* spp., Olive, Cyan, Gold, Apricot and Rose associate with a second group, and Black associate with a third group.Black asterisks indicate best association with a *Methanoperedens* genome (correlation ≥ 0.92, *P* ≤ 1 × 10^–20^); grey asterisks indicate association with a scaffold containing the *Methanoperedens* L11 marker gene (correlation ≥ 0.92, *P* ≤ 1 × 10^–20^). **c**, Frequency of genes in different functional groups in the four complete Borg genomes. **d**, Comparison of the protein family composition of Borgs and *Methanoperedens* spp. Clustering on the basis of shared protein family content highlights groups of Borg-specific protein families (blue shading) and protein families shared with their hosts (orange shading). The full clustering, including diverse archaeal mobile elements, is shown in Extended Data Fig. 5. PEGA, surface layer protein; PHA, polyhydroxyalkanoate.

**Extended Data Fig. 1:**
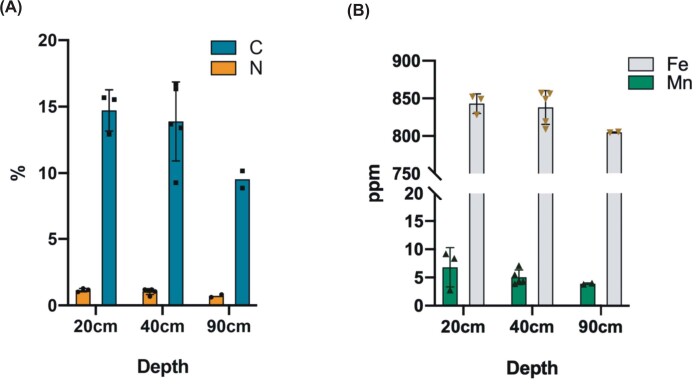
Geochemical profiles of the permanently moist and organic-rich wetland soils. (A) The mean concentrations of total carbon, nitrogen as well as (B) iron and manganese in wetland soils at 20 cm (n = 3), 40 cm (n = 5), and 90 cm (n = 2) where n denotes the number of biological samples. Deeper soils, where these extrachromosomal elements are most abundant, are somewhat depleted in carbon, iron and manganese compared to shallow soils. Error bars denote standard deviation. 36 samples were collected and sequenced, with 1 to 10 independent samples collected from the same soil depth.

**Extended Data Fig. 2:**
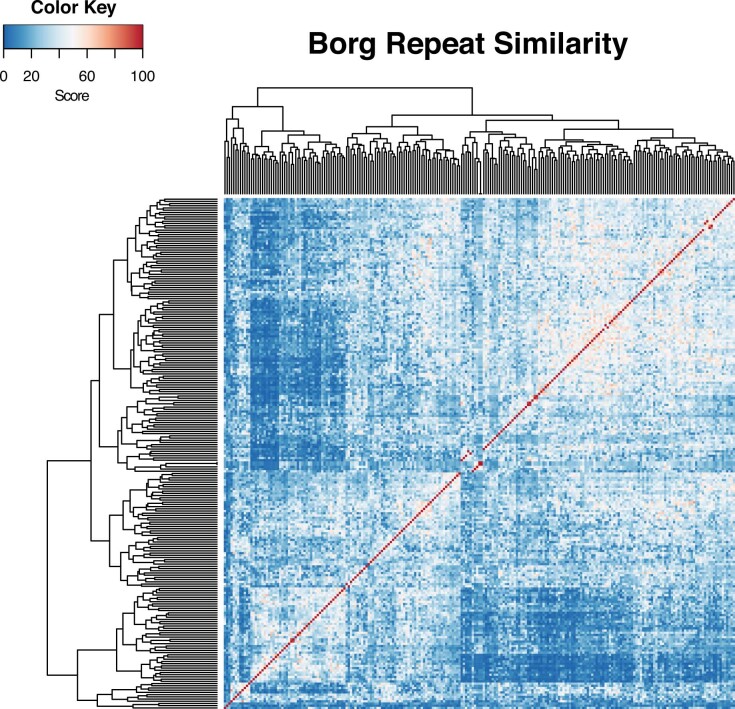
Sets of three or more perfect tandem direct repeats (TDR) are a characteristic feature of the Borg genomes. Up to 54 instances occur in the four complete Borg genomes, with, on average, one repeat every 12 (Lilac) − 31 (Sky) kbp. These repeat regions fragment assemblies and cause local assembly errors, which we resolved by manual curation (Methods). Within the TDR regions of the four curated, complete genomes, the unit repeats occur up to 20 times and unit repeats are up to 54 bps in length (Supplementary Table 2). Between 54 and 64% of these perfect TDRs are encoded in intergenic regions, although part or all of the first repeat may occur within the C- terminus of a protein-coding gene. When the TDRs occur within proteins, the unit lengths are almost always divisible by 3, so they introduce perfect amino acid repeats. TDR sequences within a single Borg genome are almost always unique. Repeat sequence comparison from the four complete curated Borgs highlights the novelty of almost all TDR sequences (both within and across genomes).

**Extended Data Fig. 3:**
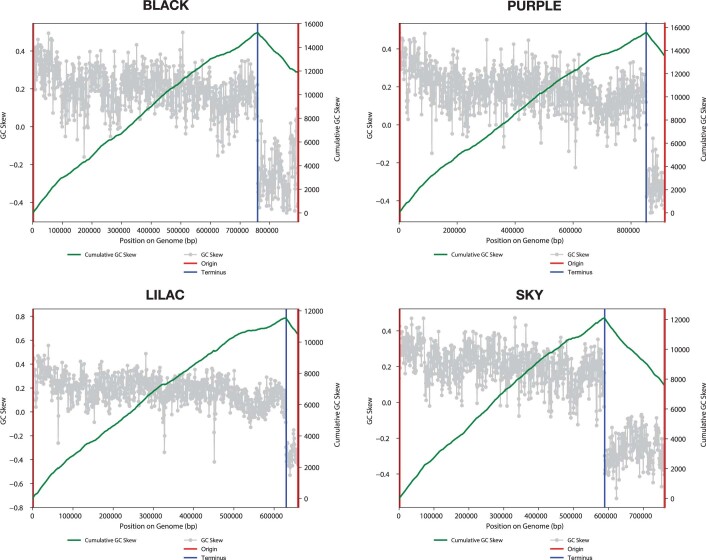
All genomes have two replichores of unequal lengths. GC skew (grey plots) and cumulative GC skew (green lines) across the four complete Borg genomes, all of which end in long inverted terminal repeats (1.4–2.7 kbp in length). The cumulative GC skew plots indicate replication is initiated in these terminal repeats (red lines). Blue lines mark the predicted replication termini. The red and blue lines define two replichores of unequal length that correspond almost completely to distinct coding strands (almost all genes on the +ve strand of the large replichore and on the -ve strand of the small replichore).

**Extended Data Fig. 4:**
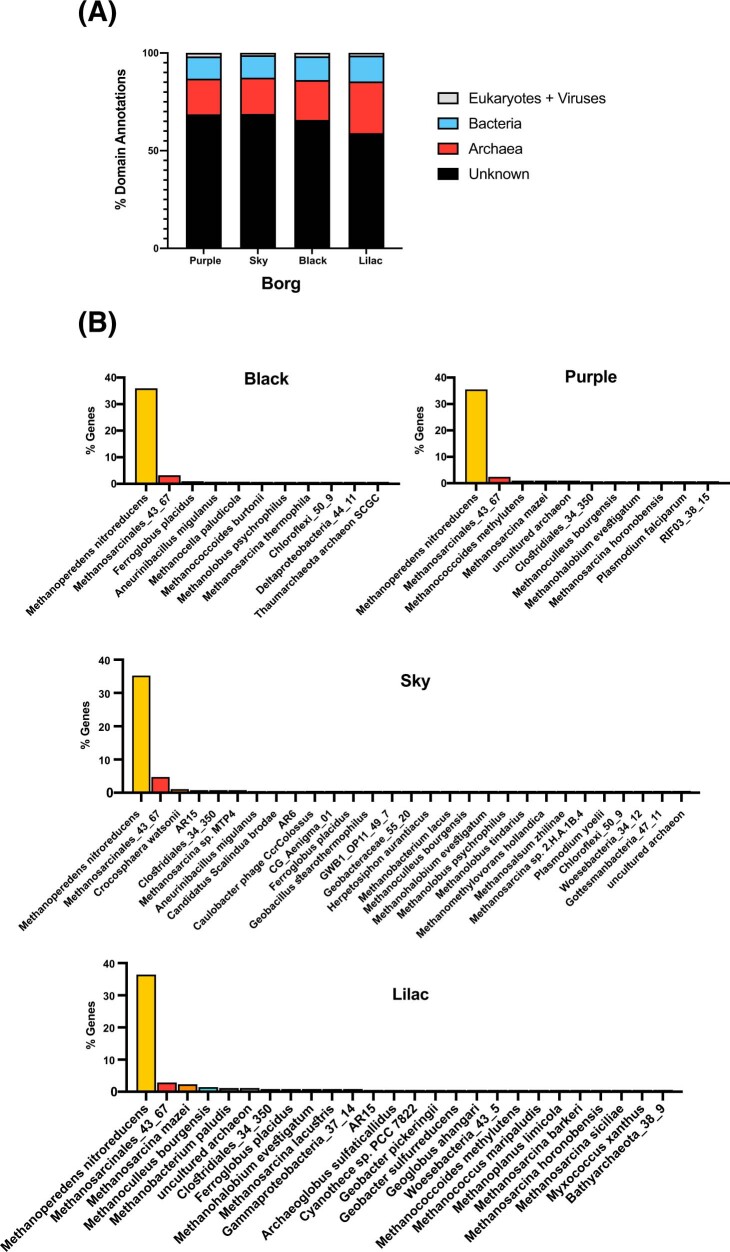
Taxonomic profiles of the four complete Borg genomes. **A**. In all cases, the majority of proteins have no similarity to proteins in the reference database (“Unknown”; e-value of > 0.0001). For the cases where a protein has an identifiable hit (blue and red bars in A), the plots in **B**. show the taxonomy of the organisms in which those hits were identified. Only cases where the same organism accounted for hits for > 0.5% of genes are shown. The results clearly indicate that the vast majority of cases where proteins have identifiable matches involve matches to proteins of *Methanoperedens* spp. (gold bars).

**Extended Data Fig. 5:**
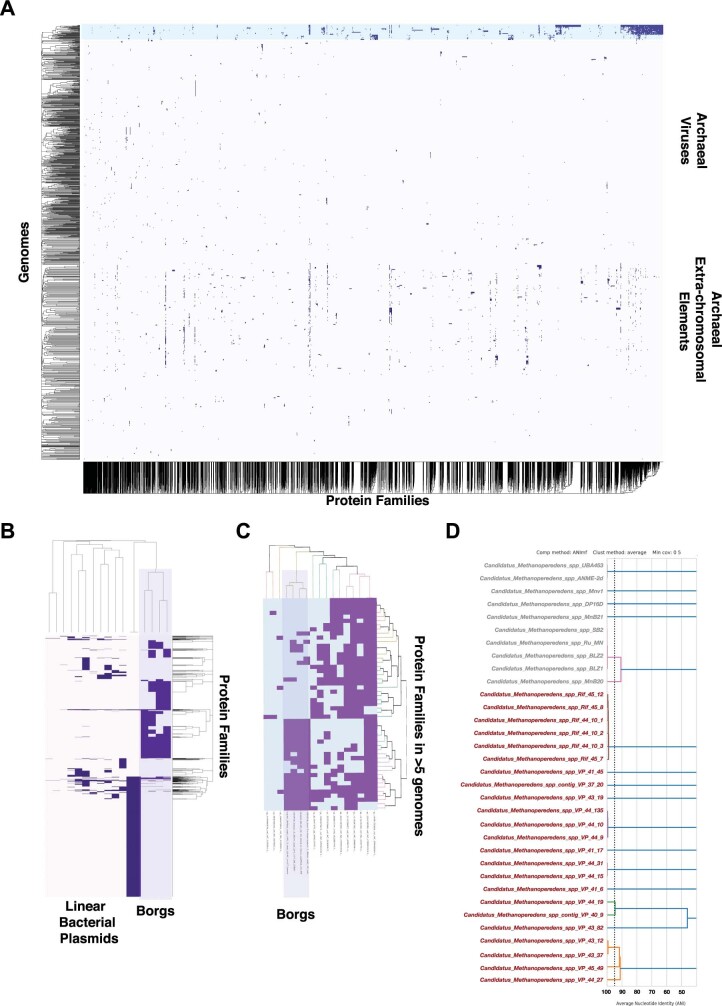
The clustering based on protein family content demonstrates that the *Methanoperedens*, Borgs, archaeal viruses and plasmids/minichromosomes are distinct from each other. (**A**) Colored blocks indicate presence of each protein family in the corresponding genome. The blue highlight at the top indicates the *Methanoperedens* spp. (top) and Borg (bottom) protein family profiles. For details see Fig. 2d. We note that archaeal plasmids are highly undersampled. If Borgs are ultimately classified as plasmids, they dramatically expand the known characteristics (e.g., size, linear genomes) and diversity of archaeal plasmids. (**B**) Borg protein inventories (purple highlight) compared to giant linear bacterial plasmids. (**C**) Protein families occurring in more than 5 genomes of Borgs and giant linear bacterial plasmids. Few protein families are shared between Borgs and linear plasmids in bacteria beyond methyltransferases, histidine kinases, and other enzymes unrelated to replication. (**D**) Average Nucleotide Identity of different *Methanoperedens* species that coexist with Borgs (red) and previously reported genomes (gray) and the 95% species threshold shown with a dashed line.

**Extended Data Fig. 6:**
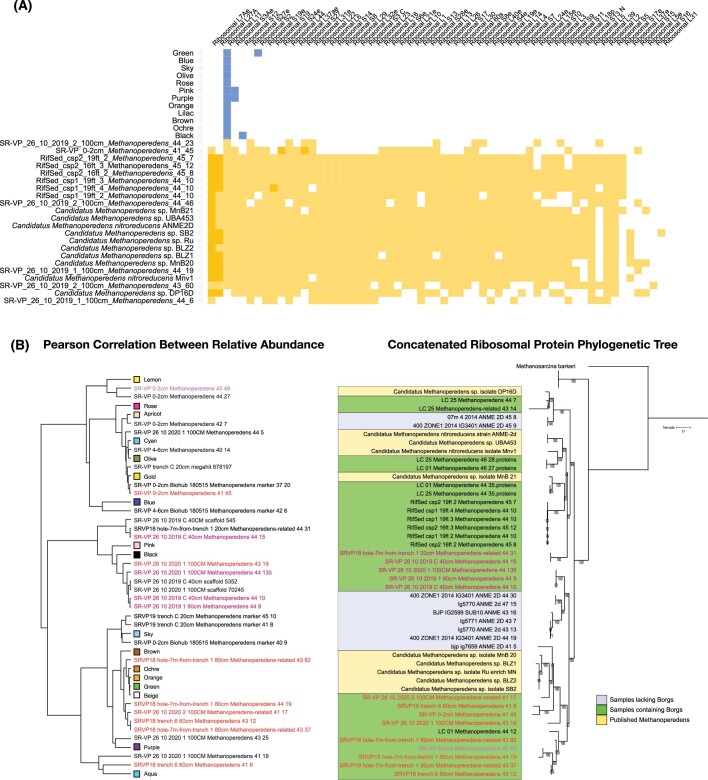
Ribosomal protein analyses and phylogenies. (**A**) The array of single-copy archaeal ribosomal genes (columns) vs. Borg (blue) and *Methanoperedens* spp. (gold) genomes illustrating that although Borgs often have rpL11 and occasionally, other ribosomal proteins, they do not have the gene inventory needed to construct ribosomes. (**B**) **Left**; Dendrogram of hierarchical clustering of all-vs-all Pearson correlation values between all Borgs and *Methanoperedens* spp. from the wetland. **Right**; Maximum Likelihood Phylogeny of concatenated ribosomal proteins from *Methanoperedens* species that do and do not coexist with Borgs and previously reported genomes. We found no data indicating the presence of Borgs in samples containing previously reported *Methanoperedens* genomes. We searched for Borgs in the samples highlighted in blue using the same methods used to detect Borgs in this study and concluded that they do not contain Borgs. A subset of the Borg-free samples contain *Methanoperedens* spp. at very high abundance levels.

By analysis of whole-community metagenomic data from wetland soils in California, USA (Extended Data Fig. 1), we discovered enigmatic genetic elements, the genomes for three of which were carefully manually curated to completion (Methods). From sediment samples from the Rifle, Colorado aquifer^15^, we recovered partial genomes from a single population related to those from the wetland soils; the sequences were combined and manually curated to ultimately yield a fourth complete genome (Methods). All four curated genomes are linear and terminated by more than 1-kb inverted repeats. The genome sizes range from 661,708 to 918,293 kb (Fig. 1a, Extended Data Table 1 and Supplementary Table 1). Prominent features of all genomes are 25–54 regions composed of perfect tandem direct repeats (Fig. 1b and Supplementary Table 2) that are novel (Extended Data Fig. 2) and occur both in intergenic regions and in genes where they usually introduce perfect amino acid repeats (Supplementary Table 2). All genomes have two replichores of unequal lengths and initiate replication at the chromosome ends (Extended Data Fig. 3). Each replichore carries essentially all genes on one strand (Fig. 1a). Although the majority of genes are novel, approximately 21% of the predicted proteins have best matches to proteins of Archaea (Extended Data Fig. 4a), and the largest group of these have best matches to proteins of *Methanoperedens* spp. (Extended Data Fig. 4b). Of note, the GC contents of the four genomes are approximately 10% lower than those of previously reported and coexisting *Methanoperedens* species (Fig. 2a). We rule out the possibility that these sequences represent genomes of novel Archaea, as they lack almost all of the single-copy genes found in archaeal genomes and sets of ribosomal proteins that are present even in obligate symbionts (Extended Data Figs. 5 and 6a and Supplementary Tables 3–6). There are no additional sequences in the datasets that could comprise additional portions of these genomes. Thus, they are clearly neither part of *Methanoperedens* spp. genomes nor parts of the genomes of other archaea.

**Extended Data Fig. 7:**
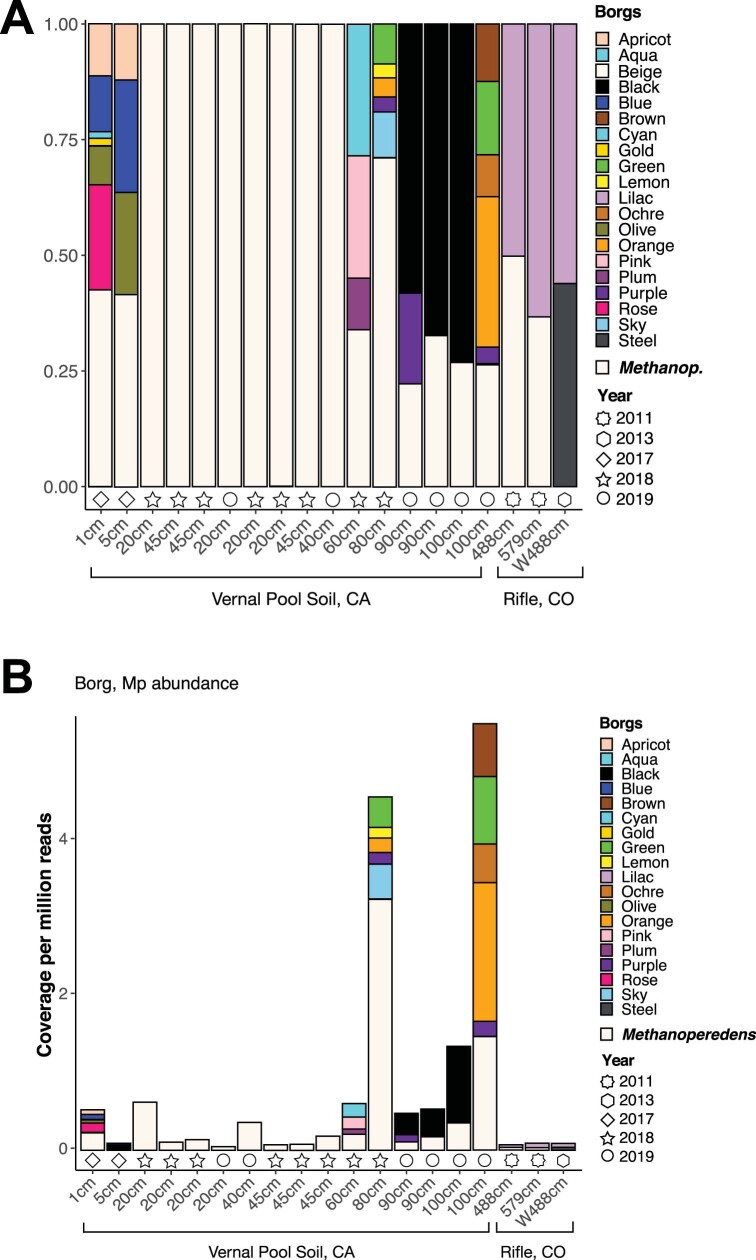
Abundance and distribution of Borgs and *Methanoperedens  spp.* in the wetland soil and Rifle aquifer. A. Relative abundances of *Methanoperedens spp*. and Borgs in samples collected over time and arrayed by sample collection depth from the wetland soils, sediments and groundwater. The absolute abundances of Borgs are far greater in the deeper compared to shallower soils B. Although some Borgs can substantially exceed all the combined abundance of *Methanoperedens  spp.*, no Borgs were detected in some *Methanoperedens*-bearing samples. “W” indicates that the sample was pumped groundwater.

Abundances of *Methanoperedens* spp. and some ECEs are tightly correlated over a set of 46 different wetland soil samples (43 genomes were included in the analysis; Extended Data Fig. 6b). This observation supports other indications that these ECEs associate with *Methanoperedens* and suggests that specific ECEs have distinct *Methanoperedens* spp. hosts (Fig. 2b). This is true for one ECE whose abundances correlate reasonably well with a specific host group, in which ECE to *Methanoperedens* spp. abundance ratios range from 2:1 to 8:1. Given their up to approximately 1-Mb length, there may be more ECE DNA in some host cells than host DNA. The Borg sequences are much more abundant in deep, anoxic soil samples (Extended Data Fig. 7a,b).

A few percent of the genes in the genomes have locally elevated GC contents that approach, and in some cases match, those of coexisting *Methanoperedens* spp. (Fig. 1b). This, and the very high similarity of some protein sequences to those of *Methanoperedens* spp., indicates that these genes were acquired by lateral gene transfer from *Methanoperedens* spp. Other genes with best matches to *Methanoperedens* spp. genes have lower GC contents (closer to those of these ECEs at approximately 33%), suggesting that their DNA composition has partly or completely ameliorated since acquisition^16^.

Archaeal ECEs include viruses^17^, plasmids^18^ and minichromosomes, sometimes also referred to as megaplasmids^10–12^. The genomes reported here are much larger than those of all known archaeal viruses, some of which have small, linear genomes^12^, and at least three are larger than any known bacteriophage^19^. These linear elements are larger than all of the reported circular plasmids that affiliate with halophiles, methanogens and archaeal thermophiles. We did not detect genes for plasmid partitioning or conjugative systems, rRNA loci or encoded viral proteins (Supplementary Table 3), and the genomes were markedly different from recently reported *Methanoperedens* spp. plasmids^20^. The distinctly lower GC content and variable copy number argue against their classification as archaeal minichromosomes^12,21^. Thus, we cannot confidently classify the ECEs as viruses, plasmids or minichromosomes. Moreover, the protein family profiles are quite distinct from those of archaeal and bacterial ECEs (Fig. 2d and Extended Data Fig. 5). Some bacterial megaplasmids have been reported to be very large and linear, but they typically encode few or no essential genes^22^, and if they contain repeats, they are interspaced (that is, not tandem)^23^. Each distinctive feature of the ECEs has been reported in microbial genomes, plasmids or viruses, but the combination of these features in these huge ECEs is unique. Thus, we conclude that the genomes represent novel archaeal ECEs that occur in association with, but not as part of, *Methanoperedens* spp. genomes. We refer to these as Borgs, a name that reflects their propensity to assimilate genes from organisms, most notably *Methanoperedens* spp.

Using criteria based on the features of the four complete Borgs, we searched for additional Borgs in our metagenomic datasets from a wide diversity of environment types. From the wetland soil, we constructed bins for 11 additional Borgs, some of which exceed 1 Mb in length (Extended Data Table 1 and Supplementary Table 1). Other Borgs were sampled from the Rifle, Colorado aquifer, discharge from an abandoned Corona mercury mine in Napa County, California, and from shallow riverbed pore fluids in the East River, Colorado. In total, we recovered genome bins for 19 different Borgs, each of which was assigned a colour-based name. We found no Borgs in some samples, despite the presence of *Methanoperedens* spp. at very high abundance levels (Extended Data Fig. 7). Thus, it appears that these ECEs do not associate with all *Methanoperedens* spp.

**Extended Data Fig. 8:**
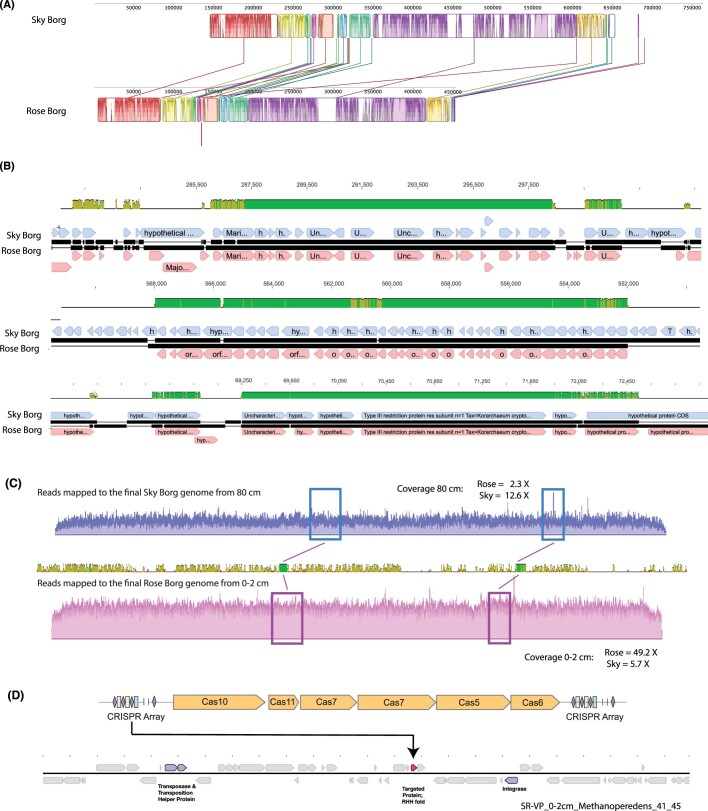
Genome comparisons and CRISPR-Cas interactions. (A) Genome-to-genome comparisons provide evidence for recombination between two of the mostly closely related Borgs, Sky and Rose. These Borgs share only moderate overall genomic nucleic acid identity although, as is the case for other Borgs (Fig. 1a), have blocks of partially alignable sequence throughout their genomes. Notable, and indicating recent homologous recombination, are 100% identical regions of up to ~11 kbp in length (B). Although not fully manually curated to completion, the relevant Rose Borg genome regions were carefully checked by inspection of the mapped reads to rule out chimeric assembly that could otherwise explain perfect identity with the Sky Borg sequence (Sky is one of the four curated complete genomes). (C) Read coverages over the Rose and Sky genomes are consistent throughout, with the regions in B noted with green boxes. (D) Diagram illustrating the organization of the Type III-A CRISPR-Cas system variant (lacking acquisition machinery and Csm6) in the Orange Borg. One spacer from the CRISPR array targets a small protein with a ribbon-helix-helix motif, a common transcriptional regulator in archaeal mobile elements, in a mobile region of a *Methanoperedens* genome bin from the same wetland site.

Pairs of the four complete Borg genomes (Purple, Black, Sky and Lilac) and three fragments of the Orange Borg are alignable over much of their lengths (Fig. 1a). The Rose and Sky Borg genomes are also largely syntenous (Extended Data Fig. 8a) and were reconstructed from different samples that contain these Borgs at very different levels of abundance. Despite only sharing a less than 50% average nucleotide identity across most of their genomes, the genomes have multiple regions that share 100% nucleotide identity, one of which is approximately 11 kb in length (Extended Data Fig. 8b,c). This suggests that these two Borgs recombined, indicating that they recently coexisted within the same host cell.

## Borg gene inventories

Many Borg genomes encode mobile element defence systems, including RNA-targeting type III-A CRISPR–Cas systems that lack spacer acquisition machinery, a feature previously noted in huge bacterial viruses^19^. An Orange Borg CRISPR spacer targets a gene in a mobile region in a coexisting *Methanoperedens* spp. (Extended Data Fig. 8d), further supporting the conclusion that *Methanoperedens* spp. are the Borg hosts.

**Extended Data Fig. 9:**
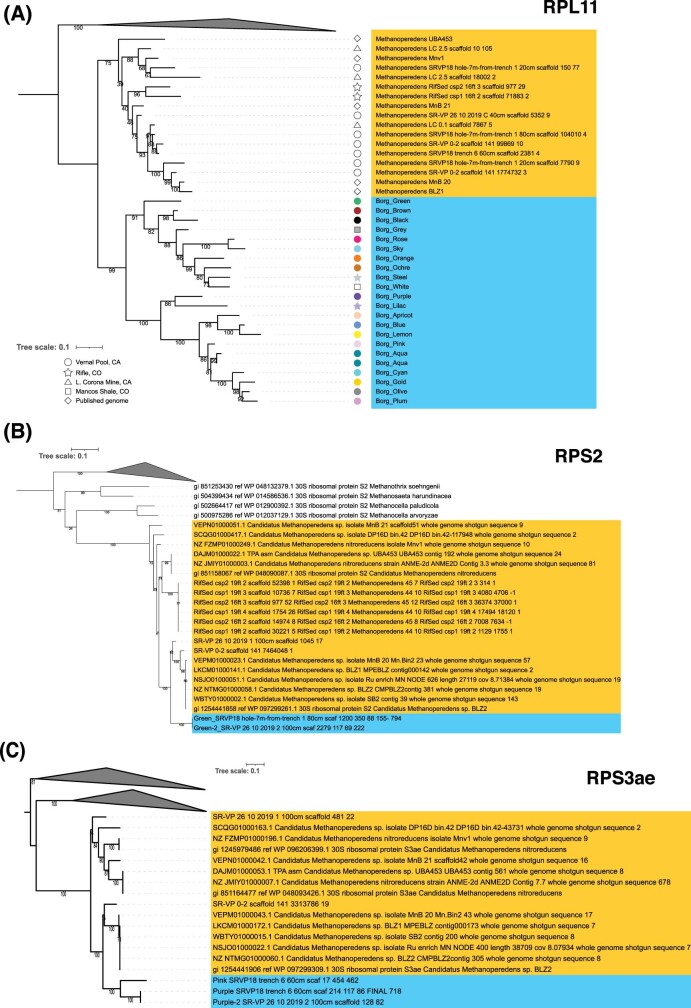
The Borg ribosomal sequences form monophyletic groups that cluster adjacent to those from *Methanoperedens* spp. Phylogenetic tree constructed using the protein sequences for (A) ribosomal protein L11 (rpL11), (B) Ribosomal protein S2 (C) Ribosomal protein 3ae.

The four complete genomes and almost all of the near-complete and partial genomes encode ribosomal protein L11 (rpL11), and some have one or two other ribosomal proteins (Extended Data Fig. 6a). The rpL11 protein sequences form a group that places phylogenetically sibling to those of *Methanoperedens* spp. (Extended Data Fig. 9), further reinforcing the link between Borgs and *Methanoperedens* spp. Four additional rpL11 sequences were identified on short contigs from the wetland group with the Borg sequences and probably represent additional Borgs (Supplementary Table 1). The topology of the rpL11 tree, and similar topologies observed for phylogenetic trees constructed using other ribosomal proteins, MCR proteins, electron transfer flavoproteins and aconitase, may indicate the presence of translation-related genes in the Borg ancestor (Extended Data Fig. 9 and Supplementary Information).

The most highly represented Borg genes encode glycosyltransferases, which are proteins involved in DNA and RNA manipulation, transport, energy and the cell surface (PEGA and S-layer proteins). Also prevalent are many genes encoding membrane-associated proteins of unknown function that may impact the membrane profile of their host (Fig. 2c). At least seven Borgs carry a *nifHDK* operon for nitrogen fixation, also predicted in *Methanoperedens* spp. genomes, and may augment the influence of the host on nitrogen cycling (Fig. 1b, Supplementary Information and Supplementary Table 6). Potentially related to survival under resource limitation are genes in at least ten Borg genomes for synthesis of the carbon storage compound polyhydroxyalkanoate (PHA), a capacity also predicted for *Methanoperedens* spp.^24^. Other stress-related genes encode tellurium resistance proteins that do not occur in *Methanoperedens* spp. genomes (Supplementary Table 5). All Borgs carry large FtsZ-tubulin homologues that may be involved in cell division, and proteins with the TEP1-like TROVE domain protein that also do not occur in *Methanoperedens* spp. genomes (Supplementary Table 5). These may form a complex similar to Telomerase, Ro or Vault ribonucleoproteins, although their function remains unclear^25^. Several Borgs encode two genes of the tricarboxylic acid cycle (citrate synthase and aconitase; Supplementary Information).

Many Borg genes are predicted to have roles in redox and respiratory reactions. The Black Borg encodes *cfbB* and *cfbC*, genes involved in the biosynthesis of F430, which is the cofactor for MCR, the central enzyme involved in methane oxidation by *Methanoperedens* spp. The similarity in GC content of Borg *cfbB* and *cfbC* and protein sequences of coexisting *Methanoperedens* spp. suggests that these genes were acquired from *Methanoperedens* spp. recently. The Blue and Olive Borgs encode *cofE* (encoding coenzyme F420:L-glutamate ligase), which is involved in the biosynthesis of a precursor for F420. The Blue and Pink Borgs have an electron bifurcating complex (Supplementary Information) that includes d-lactate dehydrogenase. Eight Borgs encode genes for biosynthesis of tetrahydromethanopterin, a coenzyme used in methanogenesis, and ferredoxin proteins, which may serve as electron carriers. The Green and Sky Borgs also encode 5,6,7,8-tetrahydromethanopterin hydro-lyase (Fae), an enzyme responsible for formaldehyde detoxification and involved in pentose-phosphate synthesis. Also identified were genes encoding carbon monoxide dehydrogenase (CODH), plastocyanin, cupredoxins and many multihaem cytochromes (MHCs). These results indicate substantial Borg potential to augment the energy conservation by *Methanoperedens* spp. This is especially apparent for the Lilac Borg.

## Host-relevant gene inventory of Lilac Borg

**Fig. 3:**
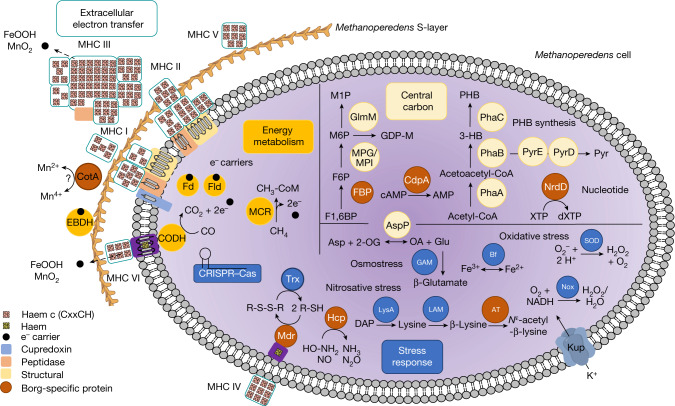
Cell cartoon illustrating capacities inferred to be provided to *Methanoperedens* spp. by the coexisting Lilac Borg. Like all Borgs, this Borg lacks the capacity for independent existence, and we infer that it replicates within host *Methanoperedens* spp. cells. Borg-specific proteins are those that were not identified in the genome of coexisting *Methanoperedens* spp. Borg-encoded capacities are grouped into the major categories of energy metabolism (including the MCR complex involved in methane oxidation), extracellular electron transfer (including MHCs) involved in electron transport to external electron acceptors, central carbon metabolism (including genes that enable production of polyhydroxybutyrate (PHB)) and stress response/defence (including production of compatible solutes). Locus codes are listed in Supplementary Table 7.

We analysed the genes of the complete Lilac Borg genome in detail as, unlike the other Borgs, the Lilac Borg co-occurs with a single group of *Methanoperedens* spp. that probably represent the host (Fig. 3 and Supplementary Table 7). Remarkably, this Borg genome encodes an MCR complex, which is central to methanogenesis and reverse methanogenesis. The *mcrBDGA* cluster shares high (75–88%) amino acid sequence identity with that of the coexisting *Methanoperedens* spp. genome. This complex is also encoded by a fragment of the Steel Borg. For both the Lilac and the Steel Borgs, the GC content of the region encoding this operon is elevated relative to the average Borg values. *Methanoperedens* spp. pass electrons from methane oxidation to terminal electron acceptors (Fe^3+^, NO_3_^−^ or Mn^4+^) via MHCs^26–28^. The Lilac Borg genome encodes 16 MHCs with up to 32 haem-binding motifs within one protein. By analogy with experiments showing that cyanophages with a photosystem gene increase host fitness, we suggest that MHC genes may increase the capacity of *Methanoperedens* spp. to oxidize methane^9,29^. However, this needs to be tested experimentally. Membrane-bound and extracellular MHC may diversify the range of *Methanoperedens* spp. extracellular electron acceptors.

The Lilac Borg encodes a functional NiFe CODH, but this is fragmented in some genomes. Other genes for the acetyl-CoA decarbonylase–synthase complex are present only in *Methanoperedens* spp. The CODH is located in proximity to a cytochrome *b* and cytochrome *c*, so electrons from CO oxidation could be passed to an extracellular terminal acceptor such as Fe^3+^ in an energetically downhill reaction. This would allow the removal of toxic CO and may contribute to the formation of a proton gradient that can be harnessed for energy conservation.

The Lilac Borg has a gene resembling the γ-subunit of ethylbenzene dehydrogenase (EBDH), which is involved in transferring electrons liberated from the hydroxylation of ethylbenzene and propylbenzene^30^. This EBDH-like protein is located extracellularly, and given haem-binding and cohesin domains, it may be involved in electron transfer and attachment.

Although the Lilac Borg lacks genes for a nitrate reductase, it encodes a probable hydroxylamine reductase (Hcp) that may scavenge toxic NO and hydroxylamine byproducts of *Methanoperedens* spp. nitrate metabolism. As the *hcp* gene was not identified in coexisting *Methanoperedens* spp., the Borg gene may protect *Methanoperedens* spp. from nitrosative stress. Proteins such as H_2_O_2_-forming NADH oxidase (Nox) and superoxide dismutase (SOD) may protect against reactive oxygen species. An alkylhydroperoxidase, two probable disulfide reductases and a bacterioferritin all may detoxify the H_2_O_2_ byproduct of Nox and SOD. The Lilac Borg also encodes genes that probably augment osmotic stress tolerance. This Borg, but not *Methanoperedens* spp., provides genes to make *N*^ε^-acetyl-β-lysine as an osmolyte. An aspartate aminotransferase links the tricarboxylic acid cycle and amino acid synthesis, producing glutamate that can be used for the production of the osmolyte β-glutamate. More importantly, perhaps, it has recently been established that a bacterial homologue of this single enzyme can produce methane from methylamine^31^, raising the possibility of methane cycling within the Borg–*Methanoperedens* spp. system.

The Lilac Borg has three large clusters of genes. The first may be involved in cell wall modification, as it encodes large membrane-integral proteins with up to 17 transmembrane domains, proteins for polysaccharide synthesis, glycosyltransferases and probably carbohydrate-active proteins. The second contains key metabolic valves that connect gluconeogenesis with mannose metabolism for the production of glycans. One gene, encoding fructose 1,6-bisphosphatase (FBP), was not identified in the *Methanoperedens* spp. genomes and may regulate carbon flow from gluconeogenesis to mannose metabolism. In between these clusters are 12 genes with PEGA domains with similarity to S-layer proteins. Cell-surface proteins, along with these PEGA proteins, account for approximately 13% of all Lilac Borg genes. We conclude that functionalities related to cell wall architecture and modification are key to the effect of these ECEs on their host, perhaps triggering cell wall modification for adaptation to changing environmental conditions (Fig. 3).

## Conclusions

Borgs are enigmatic ECEs that can approach (and probably exceed) 1 Mb in length (Extended Data Table 1). We can neither prove that they are archaeal viruses or plasmids or minichromosomes, nor prove that they are not. Although they may ultimately be classified as megaplasmids, they are clearly different from anything that has been previously reported. It is fascinating to ponder their possible evolutionary origins. Borg homologous recombination may indicate movement among hosts, thus their possible roles as gene transfer agents. It has been noted that *Methanoperedens* spp. have been particularly open to gene acquisition from diverse bacteria and archaea^6^, and Borgs may have contributed to this. The existence of Borgs encoding MCR demonstrates for the first time (to our knowledge) that MCR and MCR-like proteins for metabolism of methane and short-chain hydrocarbons can exist on ECEs and thus could potentially be dispersed across lineages, as is inferred to have occurred several times over the course of archaeal evolution^17,32^. Borgs carry numerous metabolic genes, some of which produce variants of *Methanoperedens* spp. proteins that could have distinct biophysical and biochemical properties. Assuming that these genes either augment *Methanoperedens* spp. energy metabolism or extend the conditions under which they can function, Borgs may have far-reaching biogeochemical consequences, with important and unanticipated climate implications. Confirmation that Borgs impact the rate of oxidation of methane by *Methanoperedens* and extend the conditions under which these archaea can function will require experimental evidence. This could be pursued by establishing cultures that include *Methanoperedens* with and without Borgs and comparison of the methane oxidation rates, with testing performed under a range of geochemical conditions.

## Methods

### Sampling and creation of metagenomic datasets

We analysed sequences from sediments of an aquifer in Rifle, Colorado, USA, that were retrieved from cores from depths of 5 m and 6 m below the surface^15^ in July 2011, and cell concentrates from pumped groundwater from the same aquifer collected at a time of elevated O_2_ concentration in May 2013. Discharge from the Corona Mine, Napa County, California, USA, was sampled in December 2019. Shallow pore water was collected from the riverbed at the East River, Crested Butte, Colorado sampled in August 2016. Soil was sampled from depth intervals between 1 cm and 1 m from a permanently moist wetland located in Lake County, California. Wetland soils were sampled in late October and early November 2017, 2018 and 2019. DNA was extracted from each sample (DNeasy PowerSoil Pro) and submitted for Illumina sequencing (150-bp or 250-bp reads) at the QB3 facility, University of California, Berkeley. Reads were adapter and quality trimmed using BBduk^33^ and sickle^34^. Filtered reads were assembled using IDBA-UD^35^ and MEGAHIT, gene predictions were established using Prodigal^36^ and USEARCH^37^ was used for initial annotations^34,35,37,38^. Functional predictions and predictions of tRNAs followed previously reported methods^19^.

### Genome identification, binning and curation

Hundreds of kilobytes of de novo-assembled sequences were identified to be of interest as potential novel ECEs first based on their taxonomic profile. The taxonomic profiles were determined through a voting scheme in which the taxonomy is assigned at the species to domain level (Bacteria, Archaea, Eukaryotes and no domain) by comparison with a sequence database (protein annotations in the UniProt and ggKbase: https://ggkbase.berkeley.edu/) when the same taxonomic assignment received >50% votes. Assembled sequences selected for further analysis had no taxonomic profile, even at the domain level. The majority of contigs of interest had more genes with similarity to those of archaea of the genus *Methanoperedens* spp. than to any other genus (see Extended Data Fig. 4). The second feature of interest was dominance by hypothetical proteins yet absence of genes that would indicate identification as phage or viruses or plasmids.

These initially identified large fragments were manually curated to remove scaffolding gaps and local assembly errors, to extend and join contigs with the same profile, GC and coverage, and then to extend the near-complete sequences fully into their long terminal repeats. The last step required reassignment of reads mapped at one end and at double depth to both ends. The fully extended sequences had no unplaced reads extending outwards, despite genome-wide deep coverage. Given this, and the absence of any fragments that could potentially be part of a larger genome, it was concluded that sequences represented linear genomes.

In more detail, our curation method involved mapping of reads to the de novo fragments and extension within gaps and at termini using previously unplaced reads that we added based on overlap or by the relocation of misplaced reads (these could often be identified based on improper paired read distances and/or wrong orientation). Local assembly errors were sought by visualization of the reads mapped throughout the assembly and identified based on imperfect read support, or where a subset of reads was partly discrepant and discrepancies involved sequences that were shared by tandem direct repeats of the same region (that is, the tandem direct repeat regions were collapsed during assembly). De novo-assembled sequences often ended in tandem direct repeat regions because repeats fragment assemblies. To resolve local assembly errors, gaps were inserted and reads relocated to generate the sequence required to fill the gaps. This ensured comprehensive essentially perfect agreement between reads and the final consensus sequence. In some cases, the tandem direct repeat regions had greater than the expected depth of mapped reads and no reads spanned the flanking unique sequences. In these cases, the repeat number was approximated to achieve the expected read depth, but some arrays may be larger than shown. GC skew and cumulative GC skew were calculated using iRep^39^ for the fully manually curated complete genomes, and the patterns were used to identify the origins and terminus of replication. The pattern of use of coding strands for genes (predicted in Bacterial Code 11) was compared with these origin and terminus predictions to resolve genome organization. The curated sequences were searched for perfect repeats of lengths of 50 or more nucleotides using Repeat Finder in Geneious. When repeat sequences overlapped, the unit of direct repeat was identified and the length of that repeat, number of repeats, location (within gene versus intergenic) and genome position were tabulated. Once the features characteristic of the ECEs of interest had been determined, we sought related elements. Sequences of interest were identified based on (1) credible partial alignment with the complete sequences, (2) no domain-level profile, (3) GC content of 30–35%, (4) regions with three or more direct tandem repeats scattered throughout the genome fragment, and (5) more best hits to *Methanoperedens* spp. proteins than to proteins from any other organisms. If scaffolds met criterion (1) they were immediately classified as targets. If they met most or all of the other criteria and had similar coverage values, they were binned together with other scaffolds from the same sample with these features. Often, ends of some of the contigs in the same bin overlapped perfectly and could be joined, increasing confidence in the bin quality. Genome sequences were aligned to each other using Mauve^40^. Where anomalously high (perfect) sequence identity suggestive of recent recombination was detected between Borgs, reads mapped to the region were visualized to verify that the assembly was correct (that is, not chimeric; also see information in the Extended Data).

Genome fragments were phylogenetically profiled to establish relatedness to sequences in public databases. Sequences were classified as having no detectable hit if the protein had no similar database sequence with an *E* < 0.0001.

### Correlation analyses

Reads from each sample were aligned to each *Methanoperedens* and Borg genome. Alignments were performed using bbmap^41^ using the following parameters: editfilter = 5, minid = 0.96, idfilter = 0.97, ambiguous = random. The number of reads aligning to each genome was then parsed into a matrix and the correlation between abundance patterns for *Methanoperedens* and Borg genomes was then calculated using Pearson correlation metric as implemented in scipy^42^. Correlation between a *Methanoperedens* genome and a Borg genome was deemed significant if the Pearson correlation between the two genomes was higher than 0.92. The code used for this analysis is available through Zenodo (10.5281/zenodo.6887003).

### CRISPR–Cas analysis

Borg and *Methanoperedens*-encoded CRISPR repeats and spacers were identified using CRISPRDetect^43^. The coding sequences from this study were searched against Cas gene sequences reported from previous studies^44^ using hmmsearch with *E* < 1 × 10^−5^ to identify the full locus. Matches were checked using a combination of hmmscan and BLAST searches against the NCBI nr database and manually verified by identifying colocated CRISPR arrays and Cas genes. Spacers extracted from between repeats of the CRISPR locus were compared with sequence assemblies from the sites where Borgs were identified using BLASTN-short ^45^. Matches with alignment length of more than 24 bp and 1 or less mismatch were retained and targets were classified as bacteria, phage or other. CRISPR arrays that had 1 or less mismatch, were further searched for more spacer matches in the target sequence by finding more hits with three or less mismatches.

### Protein and gene content analysis

After the identification and curation of Borg genomes and accumulation of usearch annotations for coding sequences, functional annotations were further assigned by searching against PFAM r32, KEGG, pVOG. Transmembrane regions in proteins were predicted with TMHMM. All *Methanoperedens* genomes and genome assemblies, as well as 1,153 archaeal viruses and ECEs were downloaded from the NCBI RefSeq database. Open reading frames were predicted using Prodigal, and all proteins from Borg genomes and the reconstructed ECE database were clustered into protein families and compared across genomes as previously described^19^. In brief, the coding sequences were clustered into families using a two-step procedure; first an all-versus-all sequence search was performed using an *E* value cut-off of 1 × 10^−3^, sensitivity of 7.5 and coverage of 0.5, and a sequence similarity network was built on the basis of the pairwise similarities and the greedy set cover algorithm to define protein subclusters. The resulting subclusters were grouped into protein families using a comparison of hidden Markov models. For subfamilies with probability scores of at least 95% and coverage at least 0.50, a similarity score (probability × coverage) was used as weight of the input network in the final clustering using the Markov clustering algorithm, with 2.0 as the inflation parameter. These clusters were defined as the protein families.

### Functional annotation

Genes of interest were further verified and compared using the conserved domain search in NCBI and InterproScan^46^ to identify conserved motifs within the amino acid sequence. MHCs were identified based on three or more CxxCH motifs within one gene. The cellular localization of proteins was predicted with Psort (v3.0.3) using archaea as the organism type. Proteins were compared using blastp and aligned using MAFFT^47^ v.7.407 to visualize homologous regions and check conserved amino acid residues that constitute the active site or are required for cofactor and ligand binding.

### Phylogenetic trees

For each gene, references were compiled by BLASTing the corresponding gene against the NCBI nr database, and their top 50 hits clustered by CD-HIT using a 90% similarity threshold^48^. The final set of genes was aligned using MAFFT v.7.407, and a phylogenetic tree was inferred using IQTREE v.1.6.6 using automatic model selection^49^ and visualized using iTOL^50^. Synteny plots were generated using Mauve^51^ and gene clusters through Adobe Illustrator and gggenes.

### Reporting summary

Further information on research design is available in the Nature Research Reporting Summary linked to this article.

## Online content

Any methods, additional references, Nature Research reporting summaries, source data, extended data, supplementary information, acknowledgements, peer review information; details of author contributions and competing interests; and statements of data and code availability are available at 10.1038/s41586-022-05256-1.

### Supplementary information

Supplementary Information This file contains Supplementary Fig. 1 and a Supplementary guide which includes descriptions for Supplementary Tables 1–7.

Reporting Summary

Supplementary Tables Supplementary Tables 1–7 – see Supplementary Information document for descriptions.

Supplementary Data Alignments and trees used to generate figures in the paper, in FASTA and newick format.

Supplementary Data Genome sequences generated for the paper in FASTA format.

Supplementary Data Protein sequences of the genome sequences in FASTA format.

## Figures and Tables

**Extended Data Table 1:**
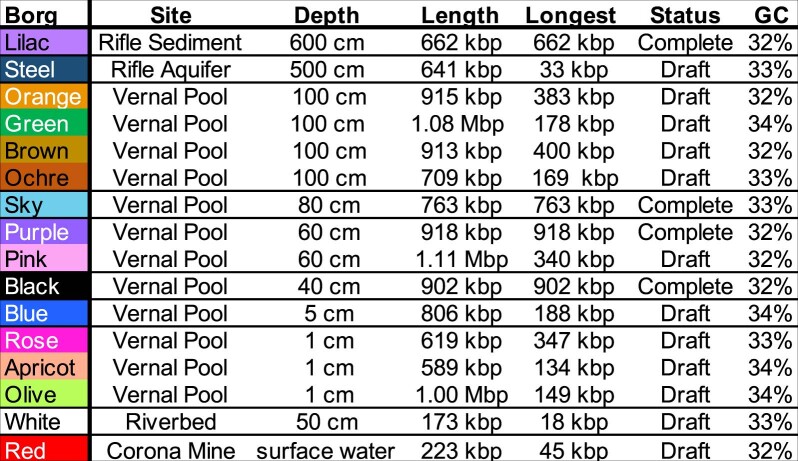
Manually curated complete and draft genomes for the best sampled Borgs

Length is the genome length. Longest is the size of the largest genome fragment. Status indicates degree of genome completeness: complete genomes have been corrected and fully verified throughout. GC is the genome-wide average GC content. For details for these and less abundant examples, see Supplementary Table 1.

## Data Availability

The Borg and *Methanoperedens* genomes and their proteins reported in this study are provided as Source Data (Supplementary Data), along with phylogenetic trees and alignments related to ribosomal protein analysis from Borgs and *Methanoperedens*. Genomes and reads can be accessed via PRJNA866293.

## Code availability

The code used to perform the correlation analysis is available through Zenodo (10.5281/zenodo.6887003). All other code is readily available at the cited sources.
