# Supplementary material for: Borgs are giant genetic elements with potential to expand metabolic capacity
Source: Nature. 2022 Oct 19;610(7933):731–6. doi: 10.1038/s41586-022-05256-1 (PMC9605863; doi:10.1038/s41586-022-05256-1)
Supplement: Supplementary file 1 — This file contains Supplementary Fig. 1 and a Supplementary guide which includes descriptions for Supplementary Tables 1–7. [file 41586_2022_5256_MOESM1_ESM.docx]

**Borgs are giant genetic elements with potential to expand metabolic capacity**

Basem Al-Shayeb, et al.

**Supplementary Information Guide**

**Supplementary Data:** FASTA files of all Borg and *Methanoperedens sp.* sequences in this study; predicted proteins; concatenated *Methanoperedens sp.* ribosomal protein tree files. Source alignments for the protein trees.

**Supplementary Table S1**: The complete, manually curated linear Borg genomes, partial genomes (bins) and bins only with short scaffolds that encode distinct Borg-like ribosomal protein L11 sequences. The Corona Mine outflow sampling site is located at 38°40’12” N, 122°32’09” W. The East River riverbed site is located at 38°55’24” N, 106°56’60” W and the Rifle CO site is located at 39°31’47” N, 107°46’20” W.

**Supplementary Table S2**: The location, sequence, length and number of tandem direct repeats (3 or more units) for the four complete Borg genomes. In cases where two direct tandem repeat regions are adjacent and involve the same repeat sequence, the numbers of tandem direct repeats in each region are separated by a comma.

**Supplementary Table S3**: Functional annotation overview.

**Supplementary Table S4-S6:** Methanoperedens-specific, Borg-specific and protein families shared between Methanoperedens and Borgs.

**Supplementary Table S7**: Gene name and description information for **Figure 3**. Sequences, specified by genome name and gene name, can be accessed via <https://ggkbase.berkeley.edu/BMp/organisms>


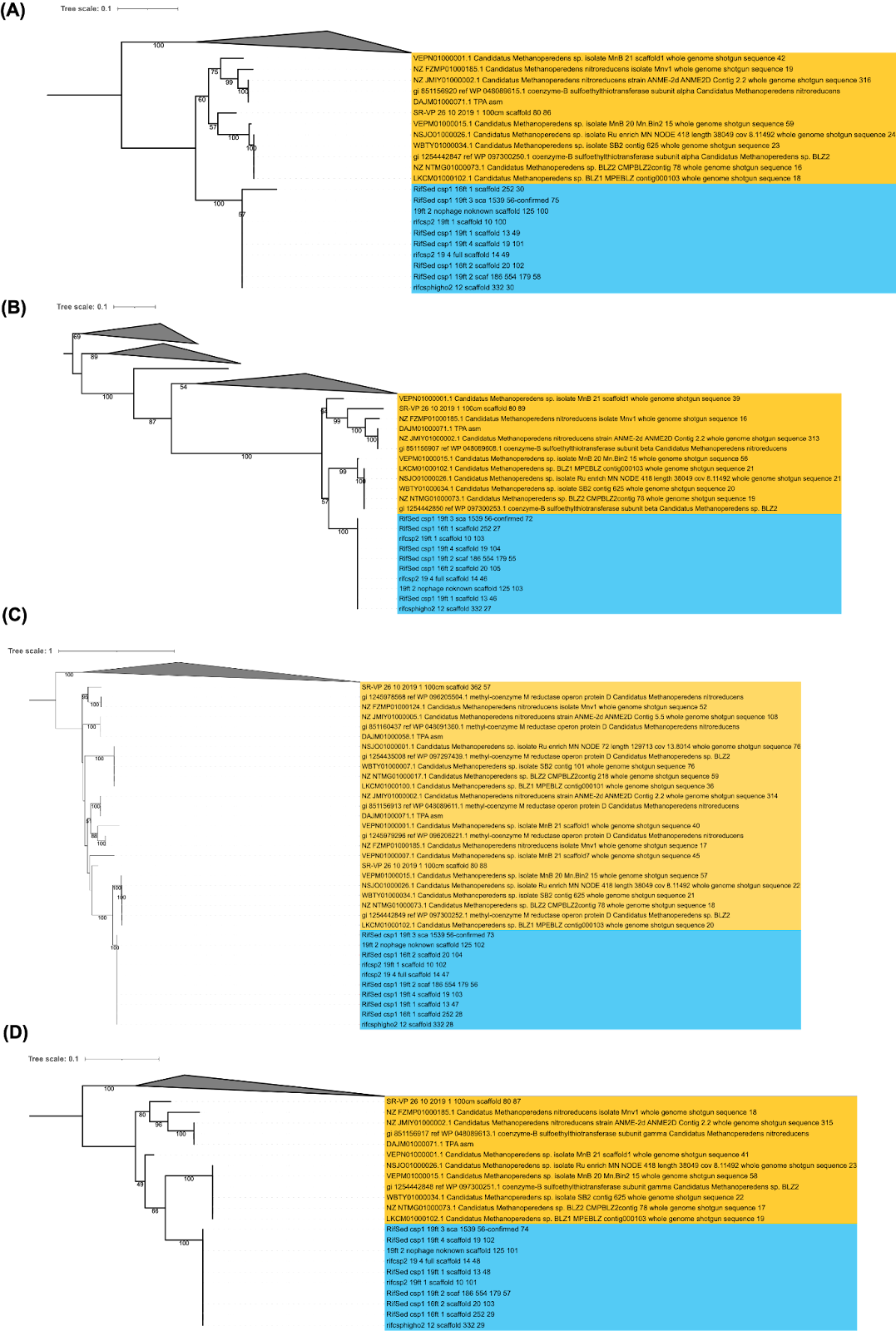
(E)


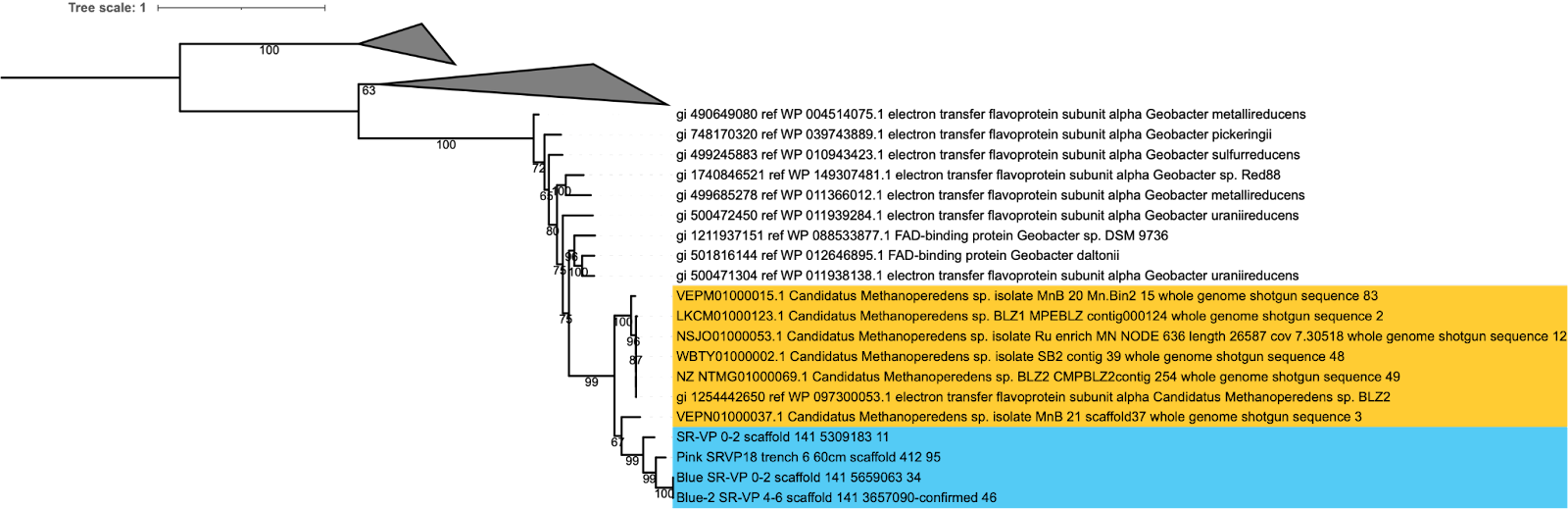


(F)


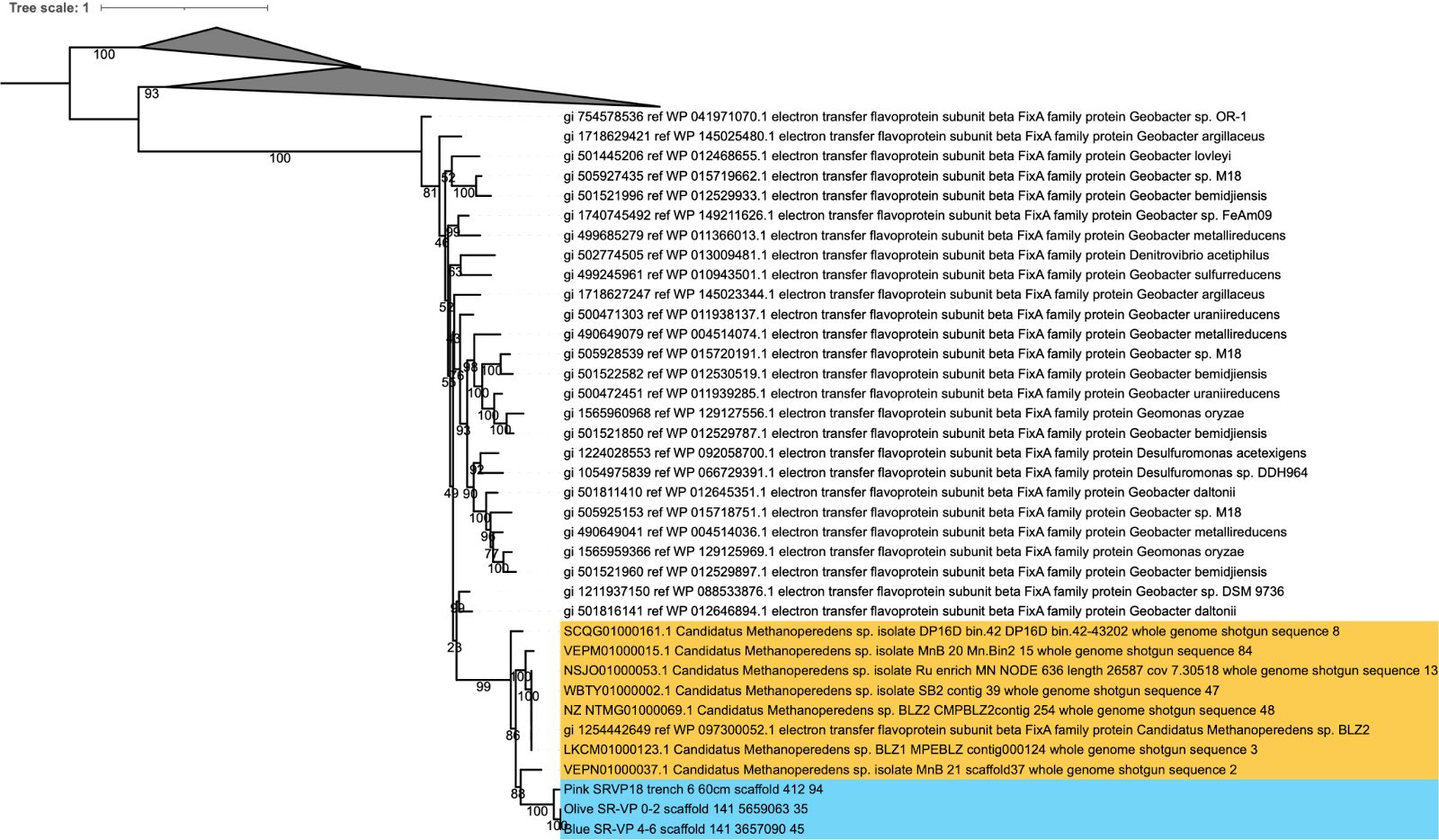


(G)
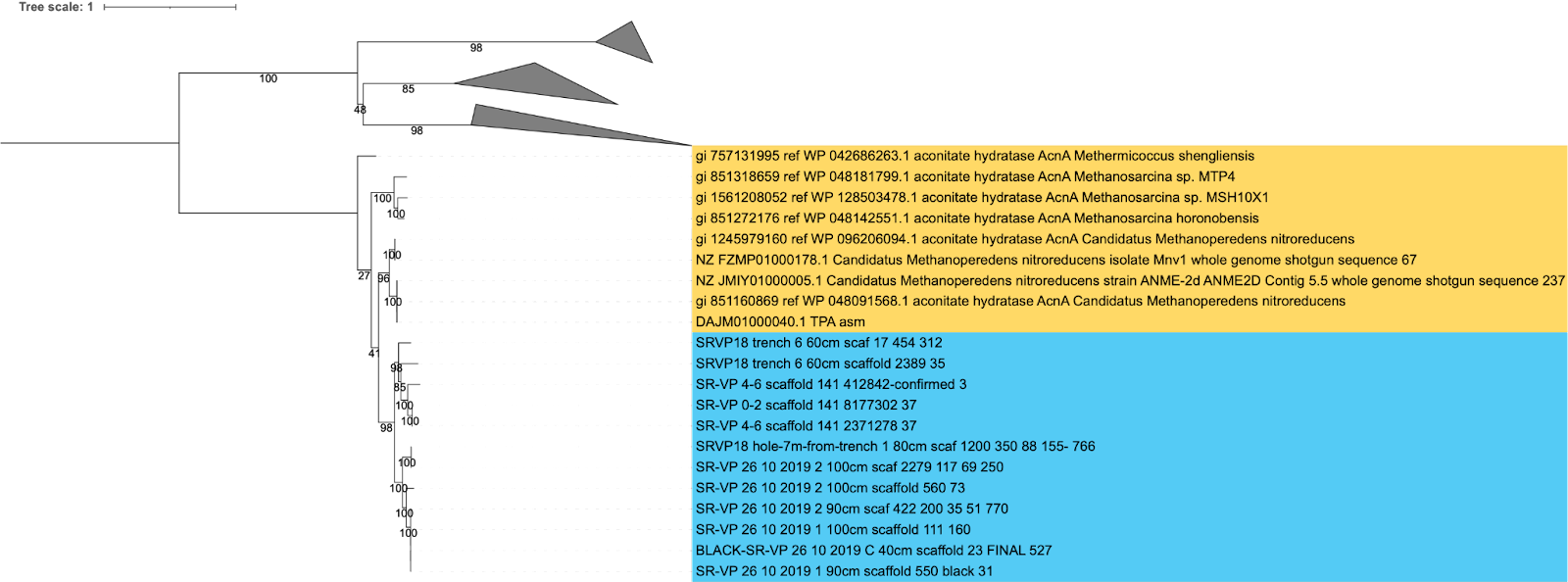


(H)
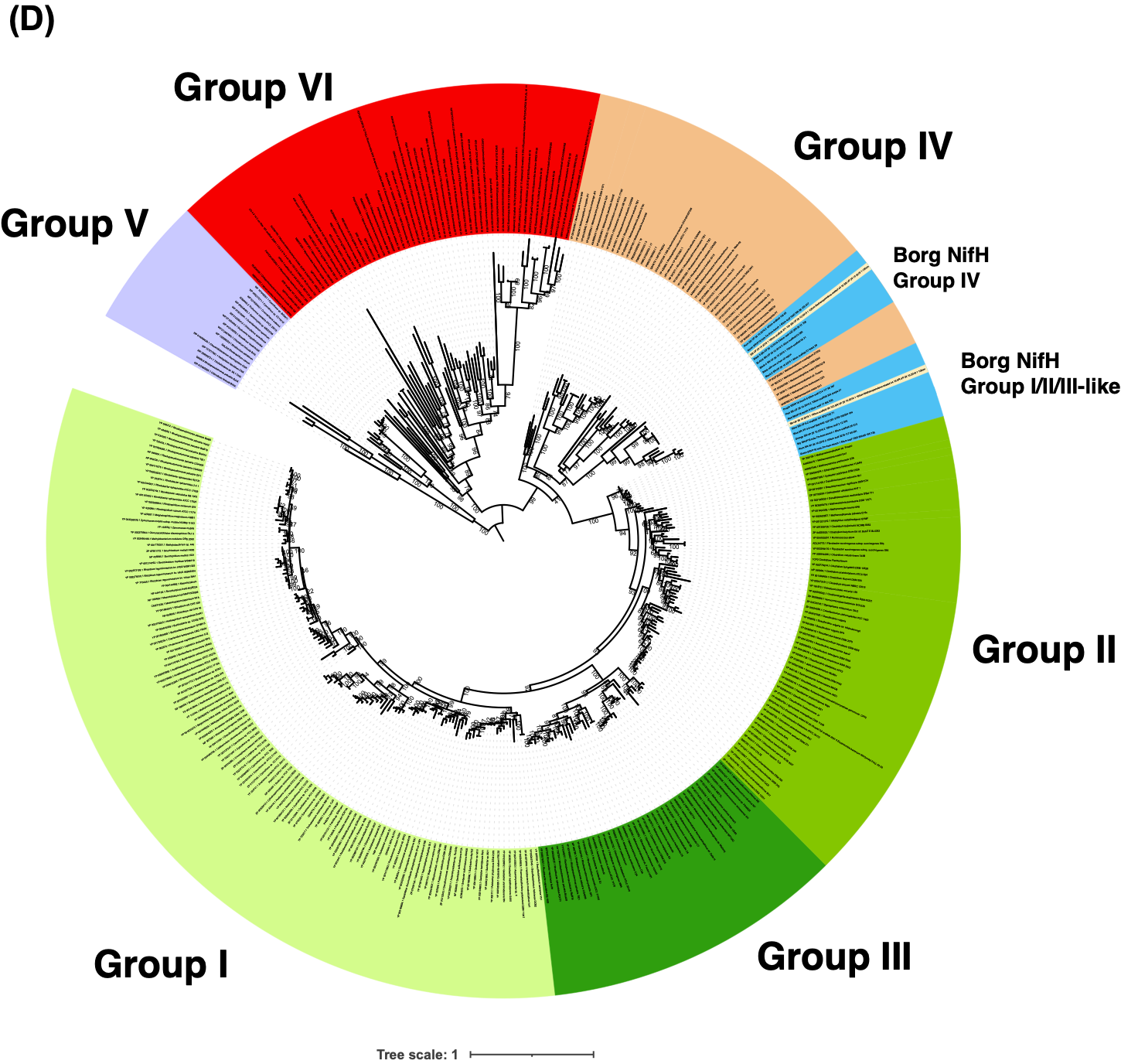


(I)


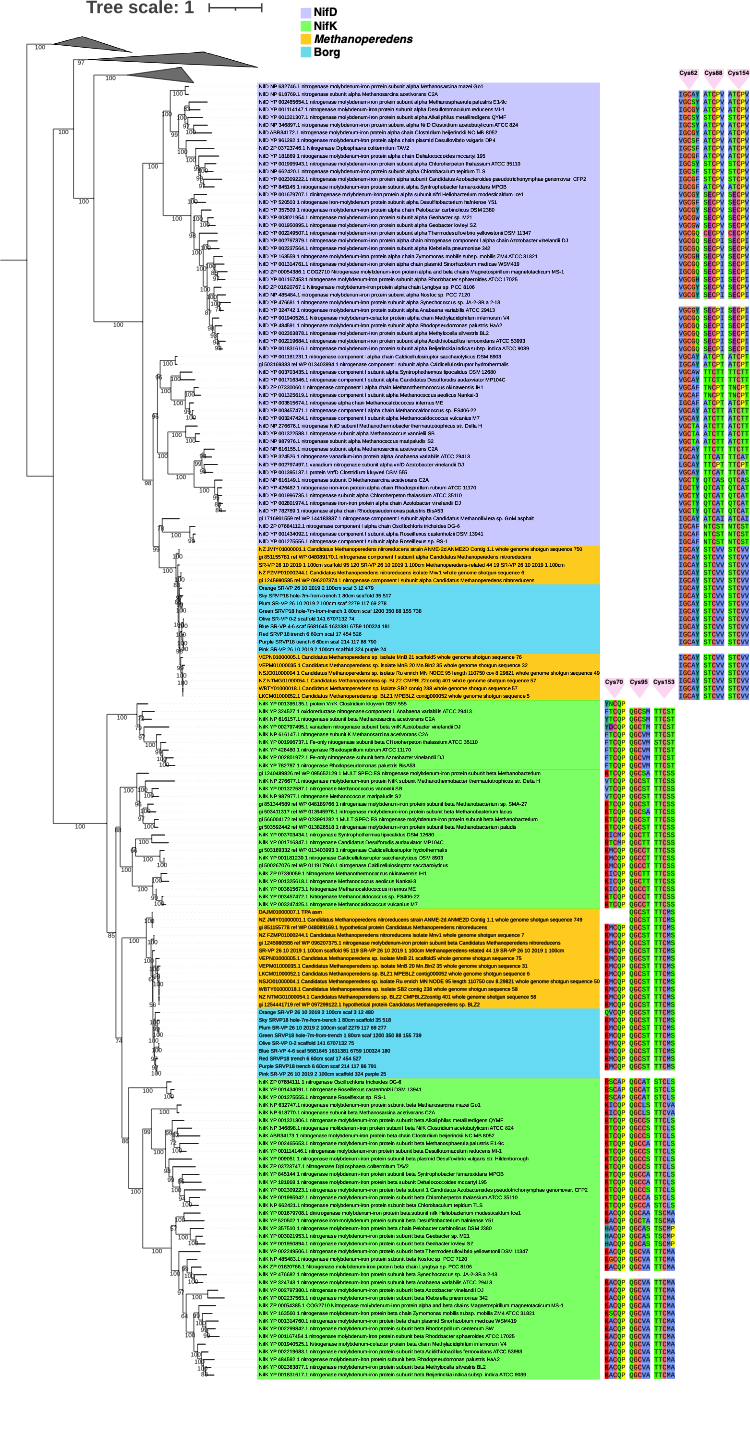


**Supplementary Figure 1**: Phylogenetic trees for key Borg genes with functional predictions showing that the protein sequences (blue) cluster sister to or within those from *Methanoperedens* (gold), with GC contents that approach those of *Methanoperedens*. (E) ETF Alpha (F) ETF Beta. (G) Aconitase (H) *nifH* and (I) *nifDK* trees with alignments showing conservation of cysteine motifs that are involved in the attachment of the P-clusters.
