# Supplementary material for: Borgs are giant genetic elements with potential to expand metabolic capacity
Source: Nature. 2022 Oct 19;610(7933):731–6. doi: 10.1038/s41586-022-05256-1 (PMC9605863; doi:10.1038/s41586-022-05256-1)
Supplement: Supplementary file 2 — Reporting Summary [file 41586_2022_5256_MOESM2_ESM.pdf]

nature portfolio

Corresponding author(s): Jillian Banfield

Last updated by author(s): Mar 3, 2022

Reporting Summary

Nature Portfolio wishes to improve the reproducibility of the work that we publish. This form provides structure for consistency and transparency in reporting. For further information on Nature Portfolio policies, see our Editorial Policies and the Editorial Policy Checklist.

Statistics

For all statistical analyses, confirm that the following items are present in the figure legend, table legend, main text, or Methods section.

n/a	Confirmed	
☐	☒	The exact sample size (*n*) for each experimental group/condition, given as a discrete number and unit of measurement
☐	☒	A statement on whether measurements were taken from distinct samples or whether the same sample was measured repeatedly
☐	☒	The statistical test(s) used AND whether they are one- or two-sided<br>*Only common tests should be described solely by name; describe more complex techniques in the Methods section.*
☒	☐	A description of all covariates tested
☒	☐	A description of any assumptions or corrections, such as tests of normality and adjustment for multiple comparisons
☐	☒	A full description of the statistical parameters including central tendency (e.g. means) or other basic estimates (e.g. regression coefficient) AND variation (e.g. standard deviation) or associated estimates of uncertainty (e.g. confidence intervals)
☐	☒	For null hypothesis testing, the test statistic (e.g. *F*, *t*, *r*) with confidence intervals, effect sizes, degrees of freedom and *P* value noted<br>*Give P values as exact values whenever suitable.*
☒	☐	For Bayesian analysis, information on the choice of priors and Markov chain Monte Carlo settings
☒	☐	For hierarchical and complex designs, identification of the appropriate level for tests and full reporting of outcomes
☐	☒	Estimates of effect sizes (e.g. Cohen's *d*, Pearson's *r*), indicating how they were calculated

Our web collection on statistics for biologists contains articles on many of the points above.

Software and code

Policy information about availability of computer code

Data collection	Geneious v9.1.8 (Licensed, paid version used in this study, free versions available)<br>BBmap v37.5<br>IDBA_UD v1.1.1<br>Bowtie2 v2.3.4.1<br>MEGAHIT v1.1.3
Data analysis	Prodigal v2.6.3<br>tRNAscan-SE v2.0<br>MMseqs2 Version: 9f493f538d28b1412a2d124614e9d6ee27a55f45<br>HHsuite v3.0.3<br>SignalP v4.1<br>DAMA v1.0<br>PSORT v3.0<br>TMHMM v2.0<br>MAFFT v7.407<br>RAxML v8.0.26<br>IQTREE v1.6.6<br>HMMER v3.1b2<br>MinCED v0.2.0<br>CD-HIT v4.8.1<br>iTOL v5<br>Mauve v2.4.0<br>InterProScan

iRep
CRISPRDetect v2.2

For manuscripts utilizing custom algorithms or software that are central to the research but not yet described in published literature, software must be made available to editors and reviewers. We strongly encourage code deposition in a community repository (e.g. GitHub). See the Nature Portfolio guidelines for submitting code & software for further information.

Data

Policy information about availability of data

All manuscripts must include a data availability statement. This statement should provide the following information, where applicable:

- Accession codes, unique identifiers, or web links for publicly available datasets
- A description of any restrictions on data availability
- For clinical datasets or third party data, please ensure that the statement adheres to our policy

All genomes are provided in the data availability statement and sequencing reads can be accessed via NCBI accessions PRJNA728365, PRJNA268031, and PRJNA441604. Sequence databases used include UniProt, ggKbase, PFAM r32, KEGG, pVOG.

Field-specific reporting

Please select the one below that is the best fit for your research. If you are not sure, read the appropriate sections before making your selection.

☒ Life sciences ☐ Behavioural & social sciences ☐ Ecological, evolutionary & environmental sciences

For a reference copy of the document with all sections, see nature.com/documents/nr-reporting-summary-flat.pdf

Life sciences study design

All studies must disclose on these points even when the disclosure is negative.

Sample size	The sample size was chosen to provide a high breadth of ecosystem coverage for the recovery of Borg genomes. Accordingly, there were no statistical methods to determine sample sizes. Data were compiled from multiple sources where Borg genomes could be found. Samples from each sampling site is listed in Table 1 and Table S1
Data exclusions	No databases or datasets were excluded during our survey.
Replication	Borg genomes were recovered from 36 independent soil samples collected in 2017, 2018, 2019, and 2020 from the same wetland field site in Lake County, CA. Up to 10 samples were collected from the same soil depth interval. DNA extracted from all samples was sequenced separately and the sequence data assembled and the datasets analyzed individually. Genomes were recovered from single samples, but in some cases the same genome was sampled independently in more than 1 sample. Sequencing reads from all samples were mapped to the most complete version of each genome to test for the presence of each genotype in each sample and to quantify the pattern of abundance of each genotype across samples. The only use of statistics was for correlation analysis that used the pattern of abundance data. The correlation analysis used a two-sided Pearson correlation test to generate a correlation metric.<br>Borg genomes were also recovered from samples collected in 2011 and 2013 from an aquifer in Rifle, Colorado. Sediment samples were taken from cores from depths of 5 and 6 m below the surface. Four replicates were collected at each depth and the genomic datasets from them were processed individually. The same Borg genotype was recovered in the 5 and 6 m depth samples and from co-assemblies. The other sites from where Borg genomes were sampled (groundwater from the Rifle site and from below the riverbed at the East River, Crested Butte, Colorado) were sampled only once. The metagenomic datasets from these samples were assembled and analyzed independently.<br><br>Host identification was verified by a combination of CRISPR targeting (sequence identity between the CRISPR spacer and Borg genome), phylogenetic analysis of ribosomal proteins, and phylum-level taxonomic profiles. Annotations were verified across multiple databases.
Randomization	After samples were collected from the natural environment they were homogenized to reduce the effects of small-scale heterogeneity, and DNA was extracted from the homogenized material. Other forms of randomization are not applicable to this study because the research relied on DNA sequences that were used to reconstruct genomes and not laboratory experiments.
Blinding	Blinding was not performed because it was not applicable to this study. We analyzed samples collected from the environment and thus the conclusions are not dependent on trial outcomes.

Reporting for specific materials, systems and methods

We require information from authors about some types of materials, experimental systems and methods used in many studies. Here, indicate whether each material, system or method listed is relevant to your study. If you are not sure if a list item applies to your research, read the appropriate section before selecting a response.

Materials & experimental systems

n/a	Involved in the study
☒	☐ Antibodies
☒	☐ Eukaryotic cell lines
☒	☐ Palaeontology and archaeology
☒	☐ Animals and other organisms
☒	☐ Human research participants
☒	☐ Clinical data
☒	☐ Dual use research of concern

Methods

n/a	Involved in the study
☒	☐ ChIP-seq
☒	☐ Flow cytometry
☒	☐ MRI-based neuroimaging
